# Malignancy prediction for calcified thyroid nodules using deep learning based on ultrasound dynamic videos

**DOI:** 10.1186/s40644-025-00944-3

**Published:** 2025-11-10

**Authors:** Tingting Qian, Yahan Zhou, Sohaib Asif, Yang Zhang, Chen Ni, Yin Zheng, Jiaheng Huang, Haoneng Shen, Renyi Zhu, Vicky Yang Wang, Dong Xu

**Affiliations:** 1 Department of Diagnostic Ultrasound Imaging & Interventional Therapy, Hangzhou Institute of Medicine (HIM), Zhejiang Cancer Hospital, Chinese Academy of Sciences, Hangzhou, 310022 China; 2 Graduate School, The Second Clinical Medical College of Zhejiang Chinese Medical University, Hangzhou, 310014 China; 3 Research Center of Interventional Medicine and Engineering, Hangzhou Institute of Medicine (HIM), Chinese Academy of Sciences, Hangzhou, Zhejiang 310000 China; 4 Center of Intelligent Diagnosis and Therapy (Taizhou), Hangzhou Institute of Medicine (HIM), Chinese Academy of Sciences, Taizhou, Zhejiang 317502 China; 5 Taizhou Key Laboratory of Minimally Invasive Interventional Therapy & Artificial Intelligence, Taizhou Branch of Zhejiang Cancer Hospital (Taizhou Cancer Hospital), Taizhou, Zhejiang 317502 China; 6 Wenling Institute of Big Data and Artificial Intelligence in Medicine, Taizhou, Zhejiang 317502 China; 7 Department of Ultrasound, Hangzhou Traditional Chinese Medicine Hospital, Hangzhou, 310000 China; 8 Postgraduate Training Base Alliance of Wenzhou Medical University, Hangzhou, Zhejiang 325035 China; 9 XianJu People’s Hospital, Zhejiang Southeast Campus of Zhejiang Provincial People’s Hospital, Affiliated Xianju’s Hospital, Hangzhou Medical College, Xianju, Zhejiang China; 10 Tongxiang First People’s Hospital, Jiao Chang Road 1918, Tongxiang, Zhejiang 314500 China; 11 Wenzhou Medical University, Wenzhou, Zhejiang 325035 China

**Keywords:** Thyroid calcified nodules, Ultrasound dynamic video, 2D static ultrasound images, Papillary thyroid carcinoma (PTC), Deep learning (DL), Calcification attention module

## Abstract

**Objective:**

The presence of calcification, especially microcalcification, is often associated with an increased risk of malignancy and closely linked to papillary thyroid carcinoma (PTC), the most common type of thyroid cancer. However, existing diagnostic ultrasound (US) imaging has critical limitations such as inability to detect subtle calcifications via standard static imaging, leading to 15–20% delayed PTC treatment or unnecessary fine-needle aspiration. This study aimed to develop a calcification-optimized, interpretable deep learning (DL) model based on dynamic ultrasound videos to determine the malignancy nature of calcified thyroid nodules.

**Design and methods:**

This study retrospectively collected ultrasound dynamic video data from 1,257 patients, containing 2,319 thyroid nodules across six hospitals between January 2020 and October 2023. Various DL models were constructed with optimization specifically implemented on the 3D InceptionResNetV2 network by including a calcification attention module to enhance sensitivity to micro-calcifications. Model performance was compared not only with those trained on 2D static ultrasound images, but also against diagnoses from four clinicians (2 junior and 2 senior radiologists). The dataset was split into training (70%, 1,623 videos), validation (10%, 232 videos), internal test (10%, 232 videos), and external test (10%, 232 videos) sets.

**Results:**

On the external test set, the optimized 3D InceptionResNetV2 model trained with dynamic videos outperformed the other four 3D DL models across all metrics: AUROC of 0.916, sensitivity of 0.860, and specificity of 0.834. Its AUROC was significantly higher than that of radiologists (0.916 versus 0.638; *p* < 0.0001). Additionally, with the assistance of the optimized model, radiologists’ diagnostic accuracy improved by 16.9% (junior) and 11.1% (senior) in the external cohort. 3D Grad-CAM further confirmed the model focused on calcified regions (consistent with clinical diagnostic logic) by generating interpretable heatmaps.

**Conclusion:**

A calcification-optimized DL model trained on dynamic ultrasound videos was proposed to efficiently and accurately predict the benign/malignant nature of calcified nodules. This tool shows promises as a non-invasive, interpretable tool for early PTC detection, supporting timely diagnosis and treatment planning.

## Key points

• This study developed a 3D InceptionResNetV2 deep learning model trained with dynamic ultrasound videos to identify and categorize calcified thyroid nodules.

• The 3D InceptionResNetV2 model was trained and tested using multi-center ultrasound video data from 1,257 patients to maximize its generalization ability in predicting the nature of calcified thyroid nodules.

• The 3D InceptionResNetV2 model, with an AUROC of 0.916, outperformed not only radiologists, but also models trained on the sole basis of static 2D images and clinical diagnoses.

## Introduction

Thyroid nodules are common endocrine disorders, with a particularly high prevalence in the adult population [1]. Epidemiological studies have shown that approximately 50% to 70% of adults may develop thyroid nodules during their lifetime; the vast majority of these nodules are classified as benign, while only about 10% to 15% are malignant [2]. Although the malignancy rate is relatively low, the presence of calcification in some thyroid nodules often triggers significant clinical concern. This is because calcification is widely recognized as an important radiological feature associated with an increased risk of malignancy in thyroid nodules [3]. Based on distinct ultrasonographic manifestations, thyroid nodule calcification can be categorized into three main types: microcalcification, macrocalcification, and mixed calcification [4]. Microcalcification refers to small, dense hyperechoic foci visible on ultrasound images [5], whereas macrocalcification refers to larger calcified areas exhibiting high echogenicity features on ultrasound images. Additionally, calcified nodules are often accompanied by other malignant features—such as irregular borders, increased nodule size, and heterogeneous internal structure—all of which can be considered malignancy indicators [6]. Therefore, the type, distribution, and relationship of calcification with other radiological features of the nodule are crucial imaging markers for determining the nodule’s malignant risk [7]. While ultrasound imaging is routinely used as the front-line diagnostic tool, given its portability and cost-effectiveness, its strong reliance on the operators’ experiences and low spatial resolution can lead to mis-interpretation and misdiagnosis. A large number of engineering studies [8] have been dedicated to developing algorithms that best utilize the speckled signals to enable delineation of nodules from US images [9].

In clinical practice, the evaluation of the benign or malignant nature of thyroid nodules relies on fine-needle aspiration (FNA) following imaging examinations. Although FNA is currently the most accurate diagnostic method, it also has several significant limitations [10]. Firstly, calcified nodules, especially those with large or dense microcalcifications, may make the aspiration procedure difficult [11]. Calcified areas are typically hard, which makes it challenging for the fine needle to accurately reach the center or malignant regions of the nodule, thereby affecting the final diagnosis of the sample [12]. Furthermore, the reliability of FNA results can be influenced by the experience of the operator, as the physician’s skill and experience directly affect the accuracy of the aspiration results [13]. Moreover, FNA is an invasive procedure, which may lead to complications such as bleeding, recurrent laryngeal nerve injury, and hoarseness [14].

Deep learning (DL) models have shown some significant advantages in predicting the benign or malignant nature of thyroid calcified nodules [15]. Wang et al. developed an AI-assisted model called the ThyroPower system, which aids in the rapid and robust cytological diagnosis of thyroid nodules [16]. Hu et al. evaluated the effectiveness of convolutional neural networks (CNNs) in predicting malignancy in thyroid nodules on ultrasound images and found that deep learning models significantly outperformed traditional methods, particularly when predicting the malignancy of calcified nodules in ultrasound images [17]. Zhang et al. proposed an attention-gated collaborative supervision network (CS-AGnet) to automatically identify calcifications, which reduced the need for pixel-level manual annotations [18]. These studies demonstrate that deep learning models can automatically extract multi-level features from ultrasound images, overcoming the limitations of traditional radiological diagnostic methods such as manual reading [19]. All aforementioned studies have demonstrated the applicability of DL models in predicting the benign or malignant nature of thyroid calcified nodules, improving diagnostic accuracy and consistency, and paving the road for personalized treatment decisions [20].

Compared to two-dimensional static images, dynamic ultrasound videos can provide more information about the features of nodules in three-dimensional (3D) space [21]. This gives dynamic ultrasound videos a significant advantage in clinical applications, particularly in the prediction of malignancy and early detection of thyroid nodules [22]. DL models can analyze and extract both local and global features from each frame in the ultrasound video, as well as the contextual relationships between frames. Based on large datasets, they can accurately predict whether a thyroid nodule has metastasized from ultrasound video data [23].

This study was set out to investigate the use of DL models for distinguishing between benign and malignant thyroid calcified nodules using thyroid video ultrasound data and assess whether the use of these models could improve the diagnostic accuracy of clinicians. Additionally, this study examined the advantages of dynamic videos over static images for diagnostic outcomes.

## Materials and methods

This study is a retrospective multicenter study using dynamic ultrasound video dataset collected from six participating hospitals. This study received ethical approval from the Ethics Committee of Zhejiang Cancer Hospital (approval number: IRB-2020-287), which served as the principal ethics approval covering all six participating centers.

### Study design and datasets

Patients with thyroid nodules who met the following inclusion criteria were included in this study: (1) aged 18 years or older; (2) underwent total thyroidectomy or unilateral thyroid lobectomy after US examination; (3) had a definitive pathological result within one month after surgery; (4) had high-quality ultrasound dynamic videos; (5) presence of calcification confirmed by either ultrasound or pathological reports (with pathological diagnosis served as the gold standard). Exclusion criteria were as follows: (1) lack of definitive pathological results and complete clinical data (e.g., missing age, thyroid disease history), or poor-quality ultrasound dynamic videos (unclear nodule boundaries or calcification features); (2) no calcified nodules observed in ultrasound videos; (3) inconsistent lesion location or size between ultrasound imaging and pathological examination; 4) received chemotherapy and/or radiotherapy (e.g., iodine-131) within 3 months prior to the ultrasound examination. This study was reported in accordance with the Standards for Reporting of Diagnostic Accuracy (STARD) 2015 statement for diagnostic research, and all 25 items of the STARD checklist are provided in the Supplementary Material.

**Fig. 1:**
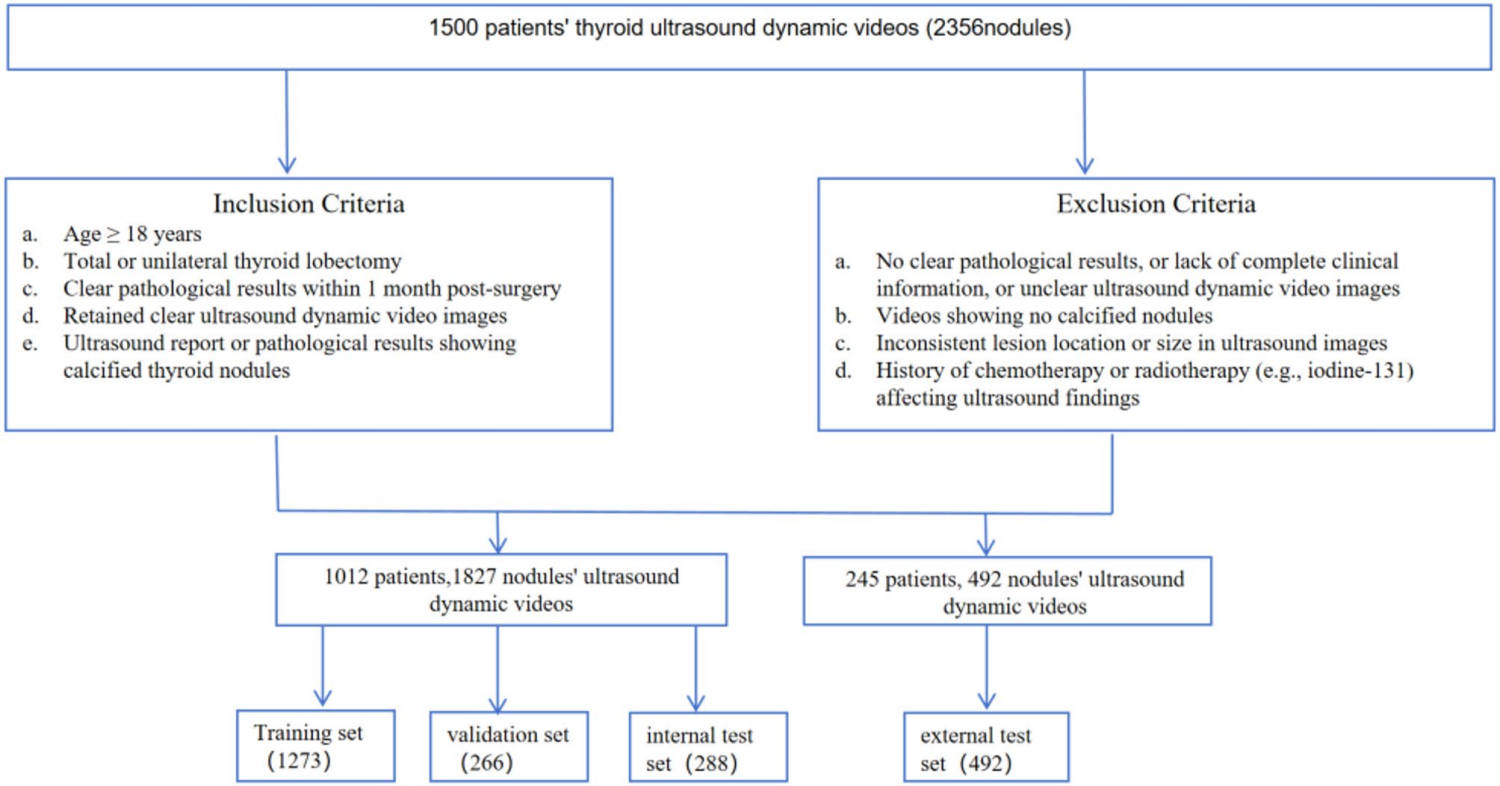
Flowchart of data collection

A breakdown of the patient numbers from each participating hospital is provided as follows. We selected 812 patients from Zhejiang Cancer Hospital between January 2020 and October 2023, and 200 patients from Jiangsu Province Hospital of Chinese Medicine in the year of 2023, totaling 1,012 patients and 1,827 ultrasound dynamic videos for model training, validation and internal testing. For the external test set, we retrospectively analyzed ultrasound dynamic video data from 245 patients (492 videos) across four hospitals between January 2023 and December 2023: Wenzhou Medical University First Affiliated Hospital (176 patients, 186 videos), PLA 63680 Unit Hospital (34 patients, 34 videos), Shenzhen Second People’s Hospital (15 patients, 15 videos), and Hangzhou First People’s Hospital (20 patients, 20 videos). There were more videos than patients because each patient might have multiple US video taken during their examination. The entire patient selection process is further illustrated in Fig. 1.

### Data preprocessing

**Fig. 2:**
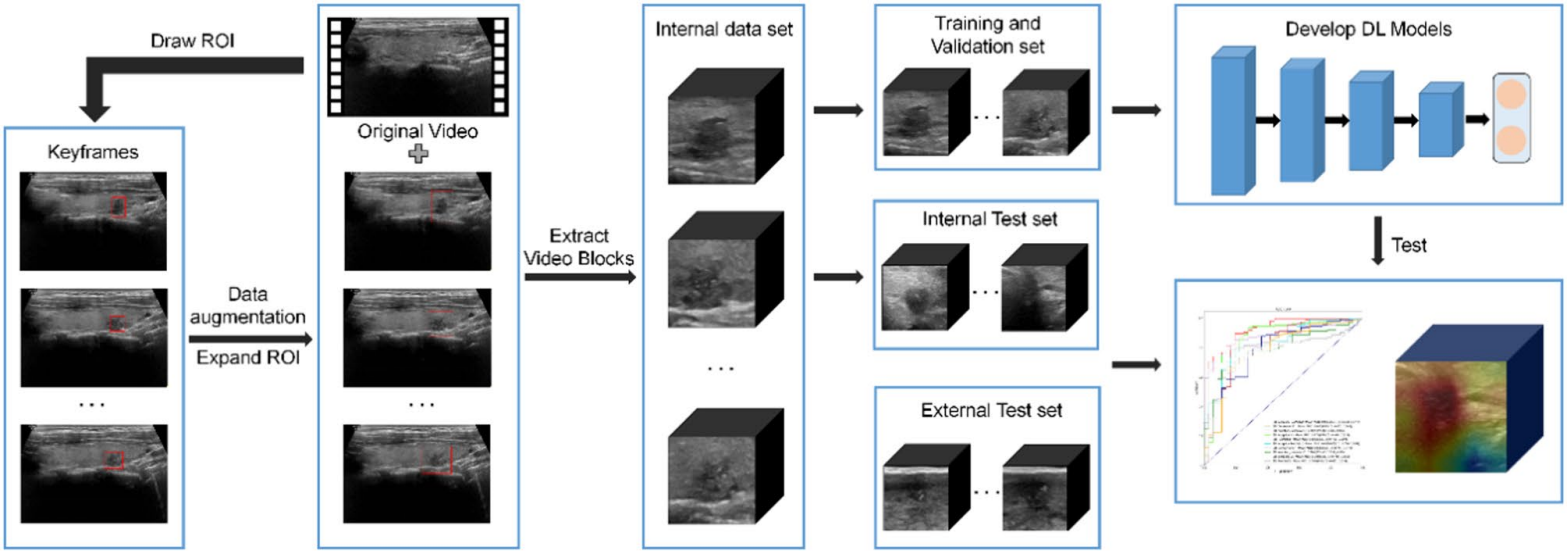
Image processing and distribution. ROI, region of interest

All thyroid ultrasound dynamic videos underwent a standardized post-processing procedure (Fig. 2). First, we used Python-based Pydicom and OpenCV packages to read the raw data stored in the Digital Imaging and Communications in Medicine (DICOM) format. Next, we outlined the nodule regions from the dynamic videos to ensure that the model could capture the key features of the nodules. Since the video lengths varied in the dataset, ranging from 44 frames to 4,126 frames, extracting the nodule regions frame-by-frame was a challenging task. Specifically, for clinicians, labeling each frame of the entire video was time-consuming and labor-intensive. Therefore, we developed an efficient labeling procedure. Clinicians were only required to select 1–10 frames from each video that clearly displayed the nodule features and marked the nodule region with a rectangular box. Then we selected 32 frames adjacent to the labeled frames as input samples for the model training. The selected frames were further cropped based on the rectangular region of interest (ROI), and resized to a final sample size of 224 × 224 × 32. It is worthwhile mentioning that we expanded the rectangular ROI by 1.5 times to enable the model to capture more information both within and around the nodule, thus enhancing the model’s robustness. This strategy not only significantly reduced labor costs but also ensured the coherence of the samples and the completeness of the nodule features. Additionally, we implemented data augmentation techniques, including random translations, cropping, and rotations, to increase the diversity of the data and reduce the risk of overfitting.

### Development of the five model

We employed five DL models—namely, InceptionResNetV2 [24], Xception [25], InceptionV3 [26], DenseNet121 [27], and ResNet50 [28]—to classify ultrasound videos and compare their performance differences. These five convolutional neural network (CNN) models, in contrast to more advanced Transformer models (which typically have larger parameter sizes and higher computational demands), offer lightweight and efficient advantages. They have been shown by prior studies to perform exceptionally well in thyroid ultrasound imaging tasks and are better suited to the practical demands of clinical environments—such as limited computational resources on bedside ultrasound devices and the need for real-time diagnostic output.

Each of the five networks underwent iterative training and backpropagation. During the training phase, video data from all patients’ cases in the training set were mixed, randomly input into the network, and validated on the validation set to identify the best parameters. In contrast, during the testing phase, we used the trained model to make predictions for all videos of the same patient case and determined the final classification of each patient using a soft voting method.

To more effectively compare the behaviour of different networks and training mechanisms on classification, this study employed the Adam optimization algorithm to standardize the hyperparameters across all experimental groups. The initial learning rate was set to 0.001, the momentum value was set to 0.9, each experiment was trained for 100 epochs, the batch size was set to 16, and the dropout rate was set to 0.5. All models in this study were implemented using Keras 2.6.0 and TensorFlow-GPU 2.6.0 frameworks on an Ubuntu 20.04 system. The host machine was equipped with an Intel(R) Core(TM) i9-13900 K @ 3.0 GHz processor and an NVIDIA GeForce RTX 3070 Ti 8GB GPU (Fig. 2).

### Statistical analysis

All statistical analyses were performed using Python (version 3.8.13), Numpy (version 1.22.3), and Scipy (version 1.8.0). Quantitative data are expressed as mean ± standard deviation (SD). The primary model performance evaluation metric is the area under the receiver operating characteristic curve (AUROC), while a 2 × 2 confusion matrix was generated to calculate accuracy (ACC), sensitivity (SEN), specificity (SPE), positive predictive value (PPV), negative predictive value (NPV), and F1 score. The receiver operating characteristic (ROC) curve was plotted to describe the true positive rate (SEN) and true negative rate (SPE). The Delong test was used to assess the significance of AUROC differences. A *p*-value < 0.05 was considered indicative of a significant difference between the two AUROCs.

## Results

### Patient characteristics

The training dataset (708 patients, 1,278 nodules) consisted of 534 females and 174 males, with approximately half of the patients aged between 18 and 45 years old and the other half aged above 45 years old. The internal testing dataset consisted of 288 thyroid nodules from 152 patients, including 112 females and 40 males, with an average age of 48 ± 13 years. Additionally, the external dataset consisted of 492 thyroid nodules from 245 patients, including 181 females and 64 males, with an average age of 50 ± 13 years (Table 1).

**Table 1:** Clinical baseline information of the patients

	Training set(708)	Validation set(152)	Internal test set(152)	External test set(245)
Gender				
Male	24.6%(174)	31.6%(48)	26.3% (40)	26.1(64)
Female	75.4%(534)	68.4%(104)	73.7% (112)	73.9%(181)
Age				
18–45	57%(403)	50%(76)	45.4%(69)	23.3%(57)
>45	43%(305)	50%(76)	54.6%(83)	76.7%(188)
Nodule location				
Left	46%(326)	46.1% (70)	46.1%(70)	44.5%(109)
Right	51.6%(365)	52% (79)	51.3%(78)	50.2%(123)
Isthmus	2.4%(17)	1.9% (3)	2.6%(4)	5.3%(13)
Pathology				
Benign	23.3%(165)	28.3% (43)	31.6%(48)	66.1%(162)
Malignant	76.7%(543)	71.7% (109)	68.4%(104)	33.9%(83)

### Performance of models

**Fig. 3:**
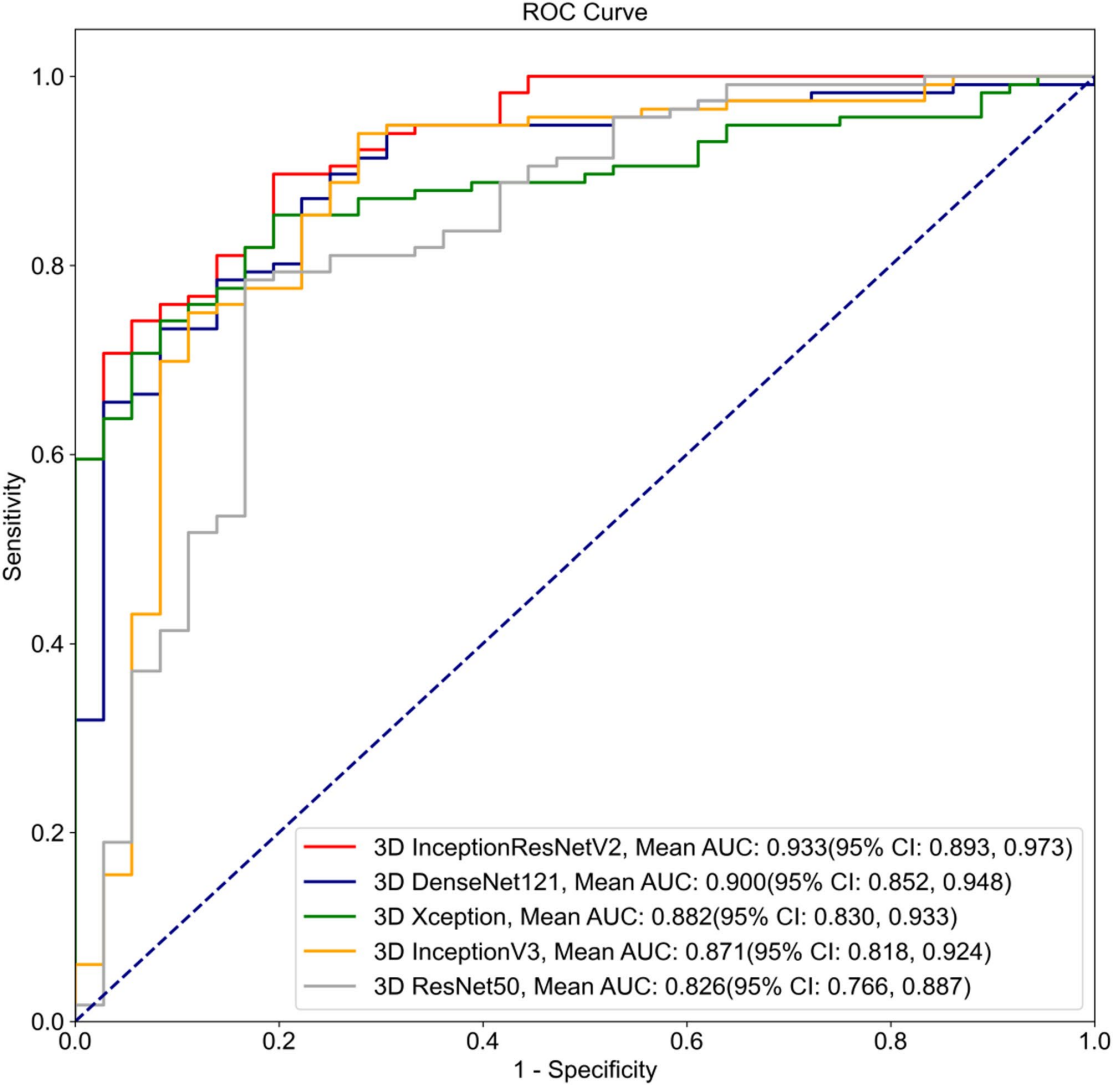
ROC curves of each model based on dynamic ultrasound video

As shown in Table 2, the 3D InceptionResNetV2 model demonstrated the following performance in the internal testing: AUROC: 0.933 (95% CI: 0.893, 0.973), accuracy: 0.855 (95% CI: 0.799, 0.911), sensitivity: 0.871 (95% CI: 0.810, 0.932), and specificity: 0.806 (95% CI: 0.676, 0.935), outperforming the other four models. 3D DenseNet had an AUROC of 0.900, while 3D Xception had an AUROC of 0.882, 3D InceptionV3 had an AUROC of 0.871, and 3D ResNet had an AUROC of 0.826 (Fig. 3).

**Table 2:** Summary of the performances of the dynamic video-based DL model using the internal test dataset. Note that due to the source of the input (i.e. US video), these models were referred to as the 3D DL models

	3D InceptionResNetV2	3D DenseNet	3D Xception	3D InceptionV3	3D ResNet
AUROC (95%CI)	0.933 (0.893, 0.973)	0.900 (0.852,0.948)	0.882 (0.830,0.933)	0.871 (0.818, 0.924)	0.826 (0.766,0.887)
Accuracy (95% CI)	0.855 (0.799, 0.911)	0.836 (0.777,0.894)	0.809 (0.747,0.872)	0.836 (0.777, 0.894)	0.783 (0.717,0.848)
Sensitivity (95%CI)	0.871 (0.810, 0.932)	0.853 (0.789,0.918)	0.802 (0.729,0.874)	0.862 (0.799, 0.925)	0.793 (0.719,0.867)
Specificity (95%CI)	0.806 (0.676, 0.935)	0.778 (0.642,0.914)	0.833 (0.712,0.955)	0.750 (0.609, 0.891)	0.750 (0.609,0.891)
PPV (95% CI)	0.935 (0.889, 0.982)	0.925 (0.875,0.975)	0.939 (0.892,0.986)	0.917 (0.866, 0.969)	0.911 (0.855,0.966)
NPV (95% CI)	0.659 (0.519, 0.799)	0.622 (0.481,0.764)	0.566 (0.433,0.699)	0.628 (0.483, 0.772)	0.529 (0.392,0.666)
F1	0.902	0.888	0.865	0.889	0.848
K (Kappa)	0.628	0.581	0.546	0.574	0.475

**Fig. 4:**
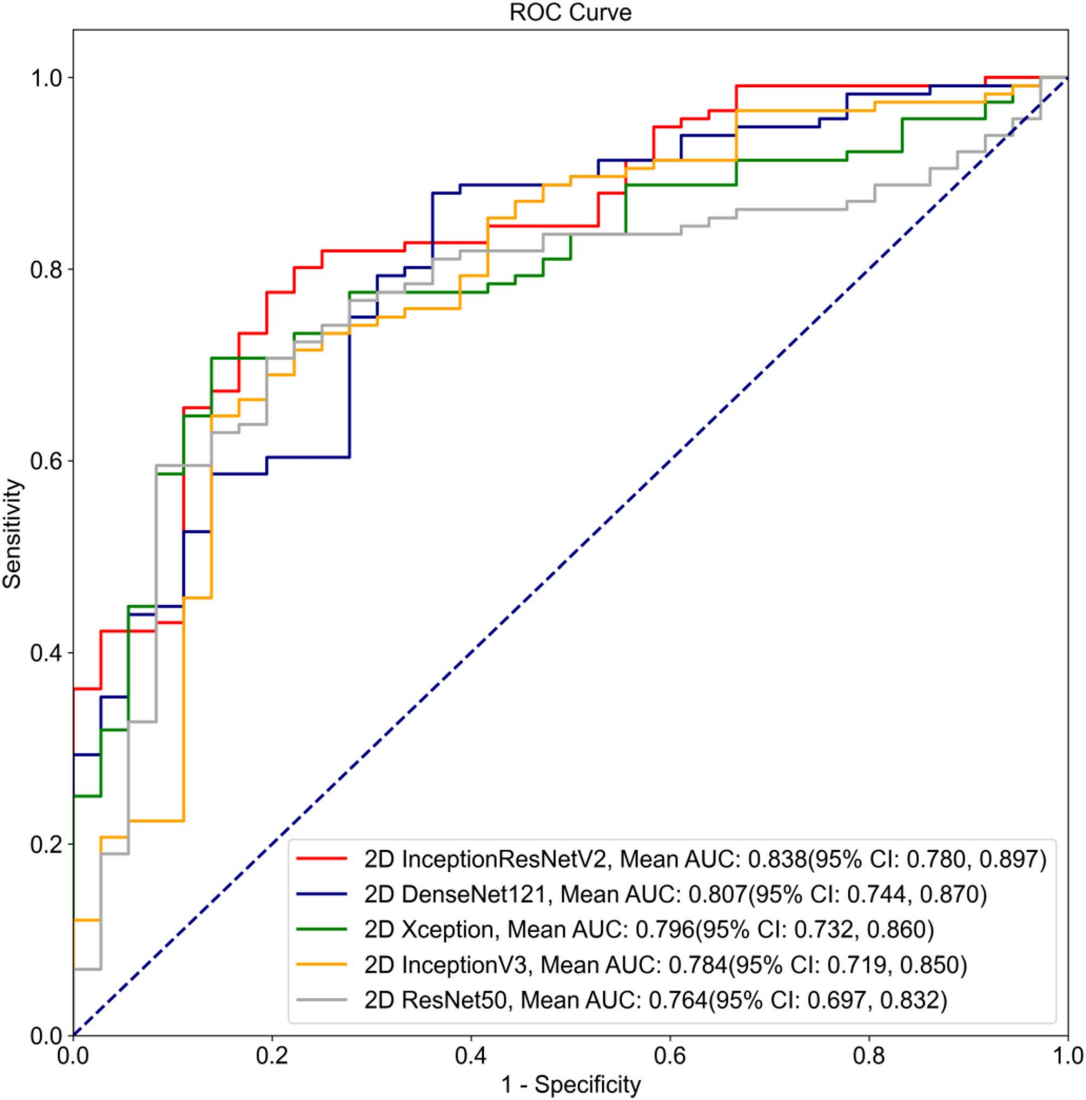
ROC curves of the models based on ultrasound static images

As shown in Table 3, the 2D InceptionResNetV2 model demonstrated the following performance in the internal testing: AUROC: 0.838 (95% CI: 0.780, 0.897), accuracy: 0.796 (95% CI: 0.732, 0.860), sensitivity: 0.802 (95% CI: 0.729, 0.874), and specificity: 0.778 (95% CI: 0.642, 0.914), outperforming the other four models (2D DenseNet, 2D Xception, 2D InceptionV3, 2D ResNet). The AUROC value of the InceptionResNetV2 model was 0.838, while 2D DenseNet had an AUROC of 0.807, 2D Xception had an AUROC of 0.796, 2D InceptionV3 had an AUROC of 0.784, and 2D ResNet had an AUROC of 0.764 (Fig. 4).

**Table 3:** Performance of the DL models based on ultrasound static images. Note that due to the source of the input (i.e. US static images), these models were referred to as the 2D DL models, in contrast to Table 2

	2D InceptionResNetV2	2D DenseNet	2D Xception	2D InceptionV3	2D ResNet
AUROC (95% CI)	0.838 (0.780, 0.897)	0.807 (0.744,0.870)	0.796 (0.732,0.860)	0.784 (0.719, 0.850)	0.764 (0.697, 0.832)
Accuracy (95% CI)	0.796 (0.732, 0.860)	0.770 (0.703, 0.837)	0.737 (0.667,0.807)	0.750 (0.681, 0.819)	0.750 (0.681, 0.819)
Sensitivity (95% CI)	0.802 (0.729, 0.874)	0.793 (0.719, 0.867)	0.733 (0.652,0.813)	0.793 (0.719, 0.867)	0.776 (0.700, 0.852)
Specificity (95% CI)	0.778 (0.642, 0.914)	0.694 (0.544, 0.845)	0.750 (0.609,0.891)	0.611 (0.452, 0.770)	0.667 (0.513, 0.821)
PPV (95% CI)	0.921 (0.868, 0.973)	0.893 (0.834, 0.953)	0.904 (0.845,0.964)	0.868 (0.803, 0.932)	0.882 (0.820, 0.945)
NPV (95% CI)	0.549 (0.412, 0.686)	0.510 (0.370, 0.650)	0.466 (0.337,0.594)	0.478 (0.334, 0.623)	0.480 (0.342, 0.618)
F1	0.857	0.840	0.810	0.829	0.826
K (Kappa)	0.507	0.434	0.399	0.369	0.390

**Fig. 5:**
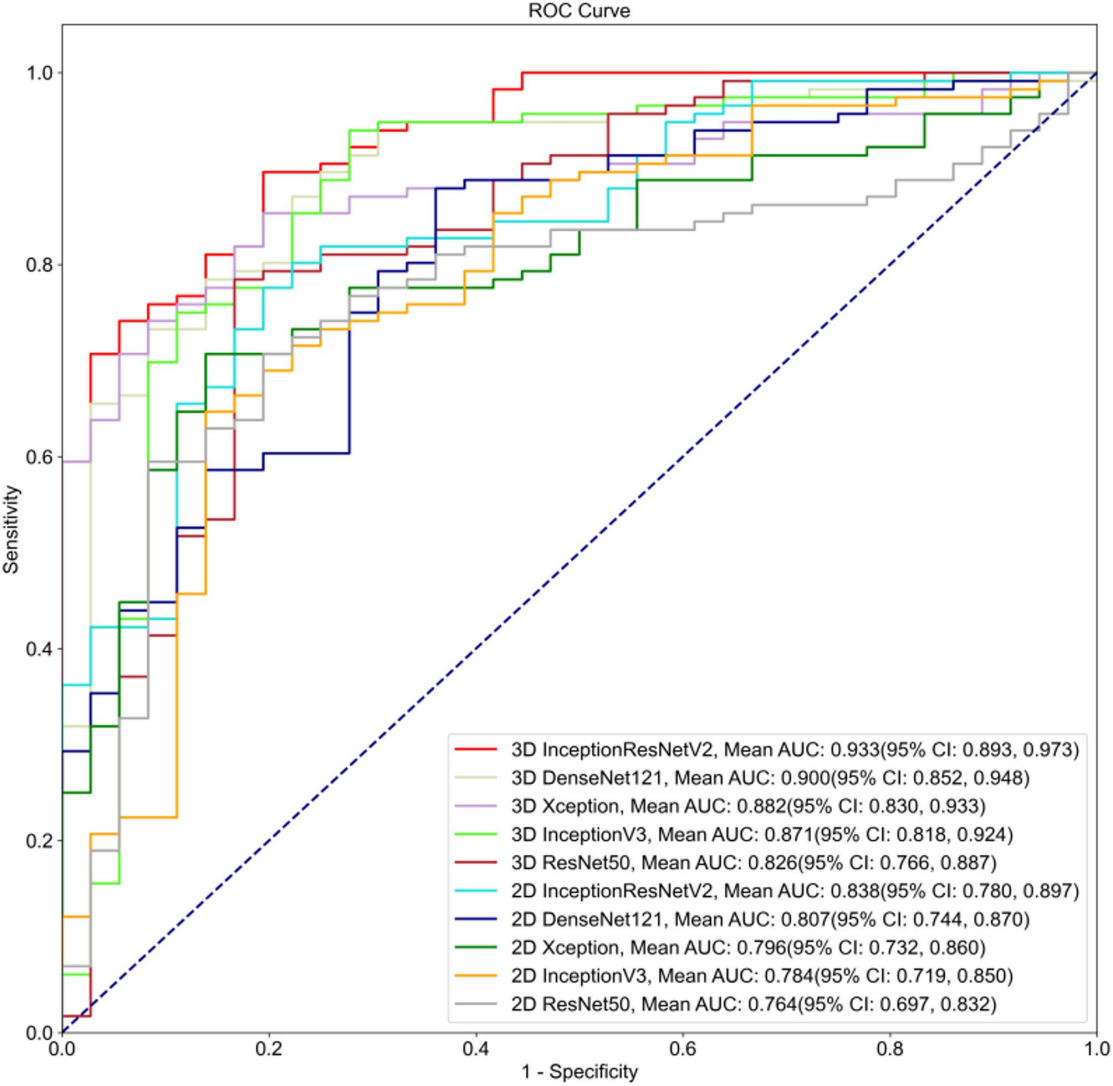
Comparative ROC curves of the models based on dynamic video and static images

To provide a more intuitive comparison of the model performance on 2D and 3D data, we plotted the ROC curves for both dynamic video and static image data. As seen in Figs. 3 and 5, InceptionResNetV2 achieved the highest AUROC, indicating that the InceptionResNetV2 deep learning model performed the best when applied to ultrasound dynamic video datasets. Subsequently, we employed the InceptionResNetV2 model to assist clinical radiologists in assessing the benign or malignant nature of thyroid calcified nodules (Fig. 5).

**Fig. 6:**
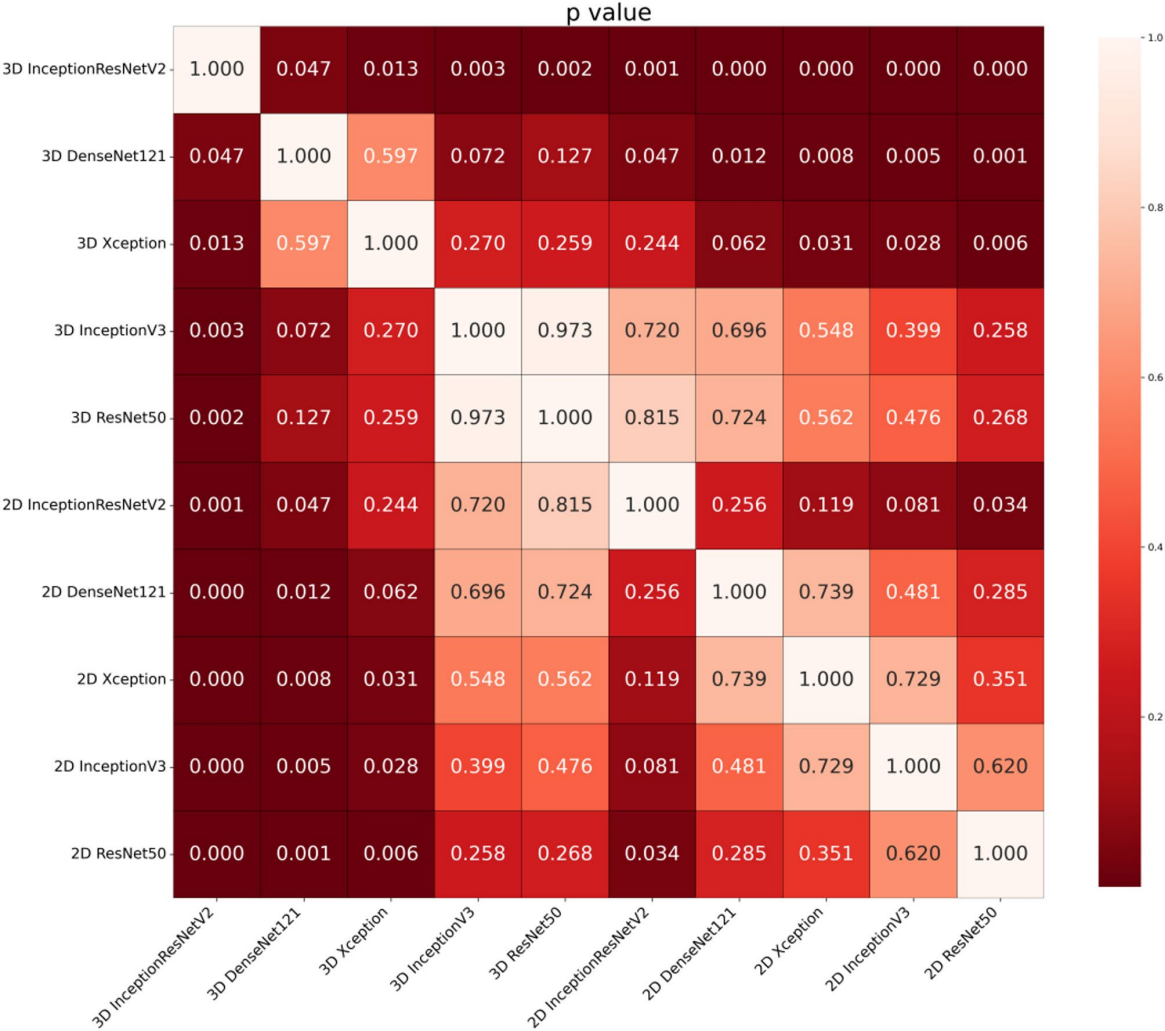
DeLong plot showing the differences between models based on dynamic video (3D) and static images (2D)

To further demonstrate the performance differences of the DL models between static images and dynamic videos, a *p*-values < 0.05 was obtained via the DeLong test for pairwise comparisons between each 3D model and its corresponding 2D counterpart, indicating that the difference in AUROC values is statistically significant (Fig. 6). In other words, the dynamic video-based models have a distinct advantage in distinguishing between benign and malignant thyroid calcified nodules.

### Diagnostic performance of the radiologists with and without the DL model assistance

**Fig. 7:**
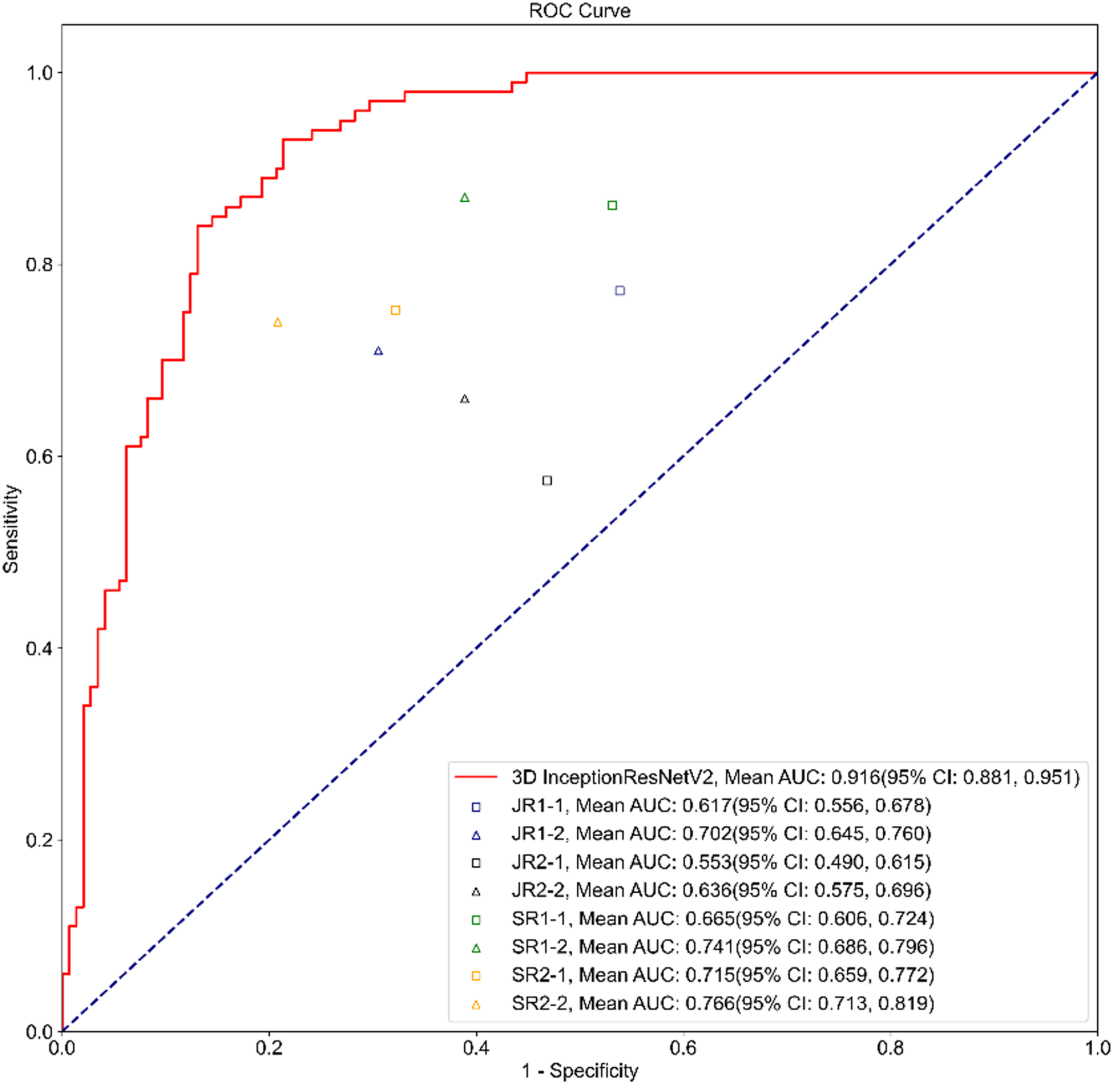
Radiologists’ diagnostic results based on ultrasound dynamic video with and without the assistance of the InceptionResNetV2 model. Note: Squares represent radiologists’ diagnostic results without the assistance of the model. Triangles represent doctors’ diagnostic results with the assistance of the model

In addition to model comparison, we also implemented readers’ study whereby clinical radiologists were asked to examine the dynamic video data from 245 patients with 492 nodules, the same dataset as the external test set. As Table 4 shows, it was observed that, regardless of whether the radiologist was junior or senior, the accuracy of the diagnostic results significantly improved with the assistance of the InceptionResNetV2 model, with a statistically significant difference (*p* < 0.05). From the ROC curve, it is evident that 3D InceptionResNetV2 still performs the best, with a significant advantage over the clinical radiologists’ results (Fig. 7).

**Table 4:** Radiologists’ ultrasound dynamic video reading results with and without the assistance of the InceptionResNetV2 model using the external dataset

	AUROC (95% CI)	Accuracy (95% CI)	Sensitivity (95% CI)	Specificity (95% CI)	PPV (95% CI)	NPV (95% CI)	F1	K (Kappa)
3D InceptionResNetV2	0.916 (0.881, 0.951)	0.845 (0.800, 0.890)	0.860 (0.792, 0.928)	0.834 (0.774, 0.895)	0.782 (0.705, 0.859)	0.896 (0.845, 0.948)	0.819	0.684
JR1-1	0.617 (0.556, 0.678)	0.590 (0.528, 0.652)	0.772 (0.690, 0.854)	0.462 (0.380, 0.543)	0.503 (0.425, 0.582)	0.742 (0.651, 0.833)	0.609	0.217
JR1-2	0.702 (0.645, 0.760)	0.701 (0.643, 0.758)	0.710 (0.621, 0.799)	0.694 (0.619, 0.770)	0.617 (0.529, 0.706)	0.775 (0.703, 0.847)	0.660	0.395
JR2-1	0.553 (0.490, 0.615)	0.549 (0.487, 0.612)	0.574 (0.478, 0.671)	0.531 (0.450, 0.613)	0.464 (0.377, 0.551)	0.639 (0.552, 0.725)	0.513	0.102
JR2-2	0.636 (0.575, 0.696)	0.631 (0.571, 0.692)	0.660 (0.567, 0.753)	0.611 (0.531, 0.691)	0.541 (0.453, 0.629)	0.721 (0.642, 0.801)	0.595	0.262
SR1-1	0.665 (0.606, 0.724)	0.631 (0.571, 0.692)	0.861 (0.794, 0.929)	0.469 (0.387, 0.550)	0.534 (0.457, 0.610)	0.827 (0.745, 0.910)	0.659	0.303
SR1-2	0.741 (0.686, 0.796)	0.717 (0.661, 0.774)	0.870 (0.804, 0.936)	0.611 (0.531, 0.691)	0.608 (0.528, 0.688)	0.871 (0.806, 0.937)	0.716	0.451
SR2-1	0.715 (0.659, 0.772)	0.709 (0.652, 0.766)	0.752 (0.668, 0.837)	0.678 (0.602, 0.755)	0.623 (0.537, 0.709)	0.795 (0.723, 0.867)	0.682	0.418
SR2-2	0.766 (0.713, 0.819)	0.770 (0.718, 0.823)	0.740 (0.654, 0.826)	0.792 (0.725, 0.858)	0.712 (0.624, 0.799)	0.814 (0.750, 0.879)	0.725	0.528

JR represents junior doctors, SR represents senior doctors. JR1-1, JR2-1, SR1-1, and SR2-1 represent the diagnostic results of doctors without the assistance of the model. JR1-2, JR2-2, SR1-2, and SR2-2 represent the diagnostic results of doctors with the assistance of the model

**Fig. 8:**
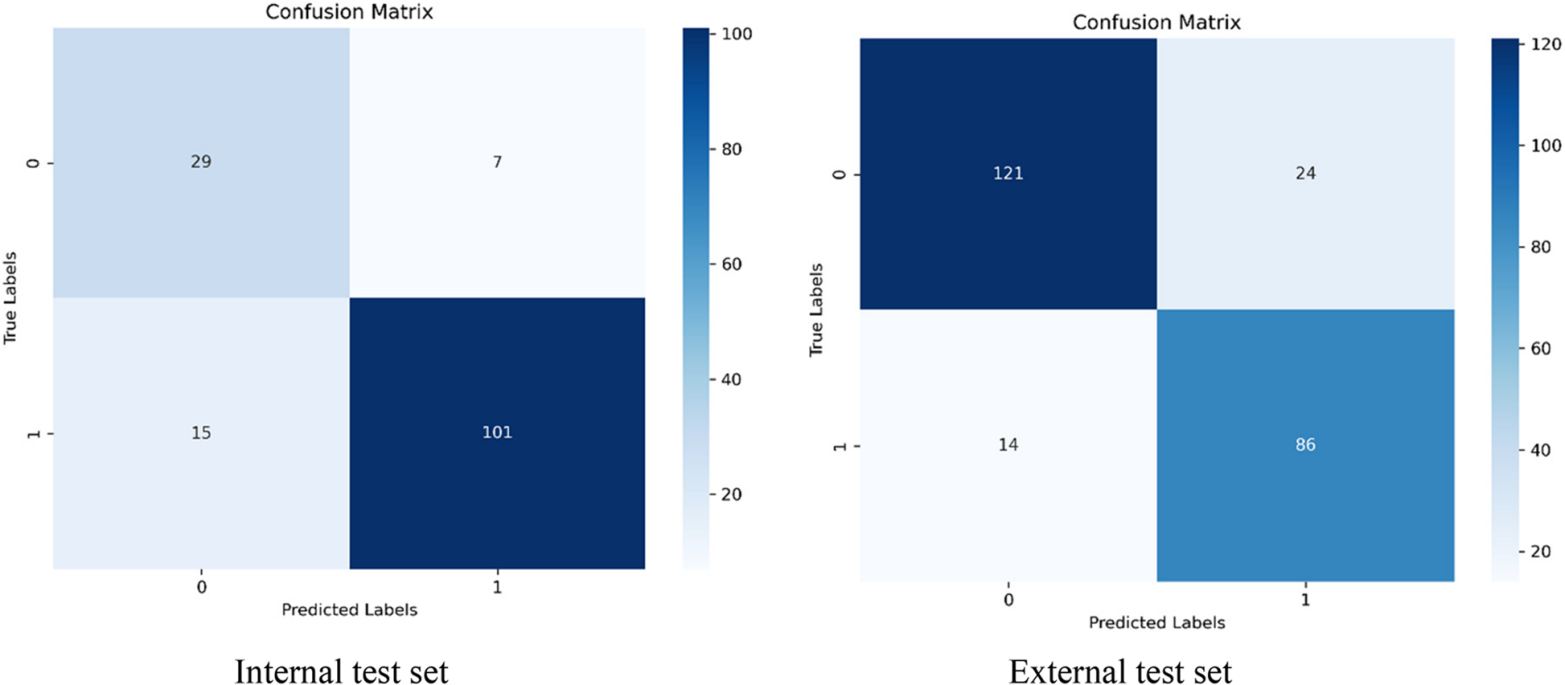
Confusion matrices for the internal and external test sets

The accuracy of the internal test set was 85.5%, while the accuracy of the external test set was 84.5%. As expected, the performance of the external test set has decreased but only by 1%. Compared to the internal test set, the model’s performance on the external data has declined, indicating that the model may have a weaker generalization ability for external data (Fig. 8).

**Fig. 9:**
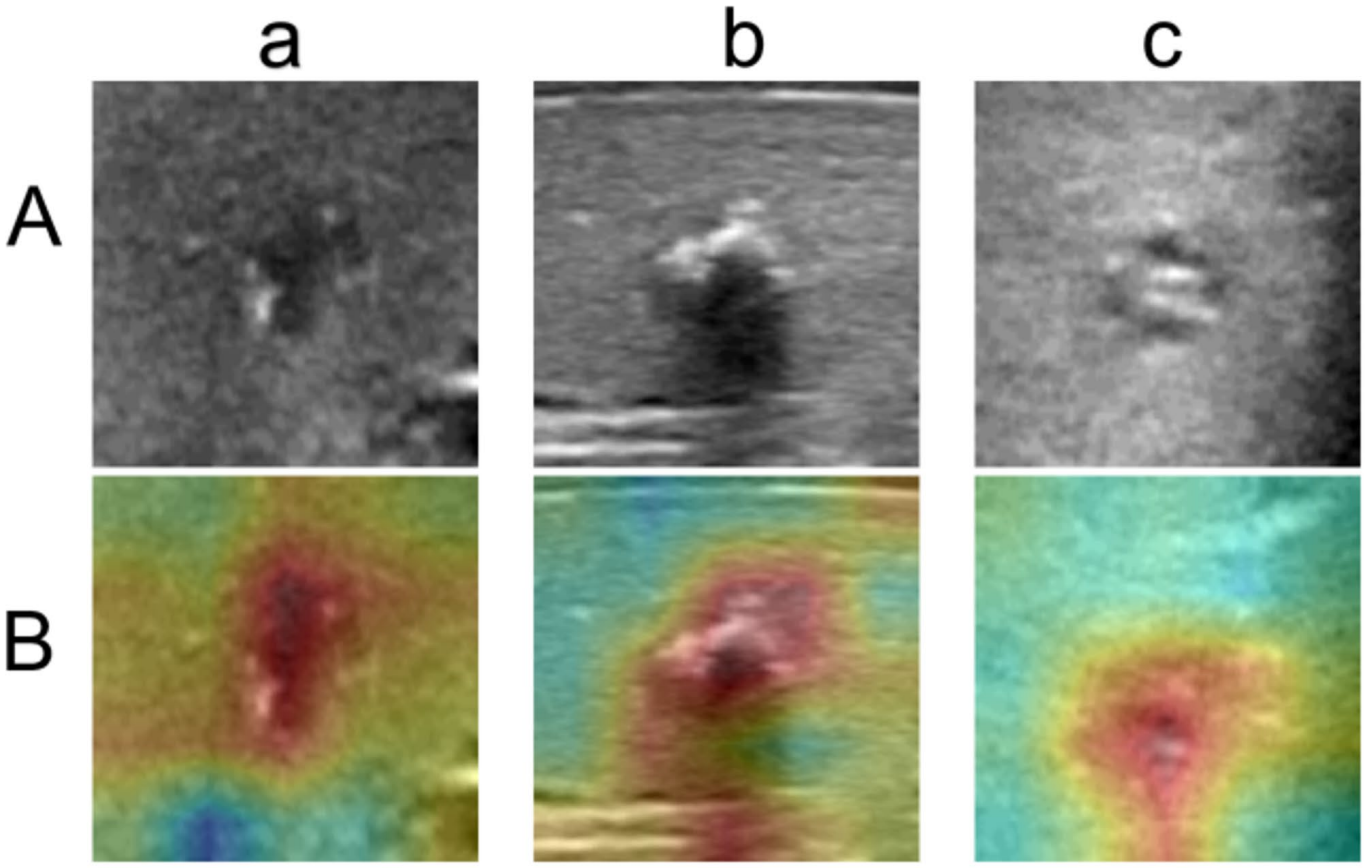
The ultrasound images of thyroid calcified nodules from three different patients (a, b, and c) are presented, along with their corresponding heatmaps

In both US images (Row A) and heatmaps (Row B), white areas indicate the highest level of calcification identified by the model while the red part highlights the location of the nodule. This color distribution helps clarify the strength, extent, and contrast of each nodule’s calcified area with the surrounding tissue, providing a clear visualization of the nodule’s calcification characteristics (Fig. 9).

## Discussion

Thyroid calcified nodules are generally considered to be closely associated with PTC, one of the common types of thyroid cancer. This study explored the application of deep learning (DL) models in predicting the benign and malignant nature of thyroid calcified nodules, particularly focusing on the use of dynamic ultrasound videos. Through a retrospective analysis of ultrasound dynamic video data from 1,257 patients (1,012 internal + 245 external) with a total of 2,319 thyroid nodules, we found that combining dynamic ultrasound videos with DL models, especially the 3D InceptionResNetV2 model (optimized with a calcification attention module), significantly improved the accuracy of predicting the benign and malignant nature of thyroid calcified nodules. The optimized 3D InceptionResNetV2 achieved an AUROC of 0.916, sensitivity of 0.860, and specificity of 0.834 on the external test set. For the internal test set, 2D InceptionResNetV2 achieved an AUROC of 0.838, sensitivity of 0.802, and specificity of 0.778. DL models based on dynamic ultrasound videos outperformed those based on static images in all evaluation metrics (AUROC, sensitivity, specificity), which is of great significance for reducing misdiagnoses and missed diagnoses of thyroid calcified nodules in clinical practice, especially for tiny calcifications (≤ 1 mm).

Previous studies have shown that calcification characteristics are important markers for malignant thyroid nodules, especially in ultrasound examinations, where microcalcifications are often closely linked to the occurrence of papillary thyroid carcinoma, with an incidence of 35–50% in PTC cases [3, 29]. However, traditional imaging methods, such as static images and fine needle aspiration (FNA), often face challenges when dealing with calcified nodules: static images miss dynamic malignant signs (e.g., calcified particle movement), and FNA is invasive with a 15–20% unnecessary sampling rate [30]. In this study, we proved that dynamic ultrasound videos offer a non-invasive alternative for diagnosing thyroid calcified nodules, potentially reducing the need for FNA.

The results of this study further demonstrate the potential of combining deep learning with dynamic videos can overcome the aforementioned issues and improve the accuracy of clinical diagnosis. In assessing the nature of thyroid calcified nodules, the diagnostic results of four radiologists (2 junior, 2 senior) improved with the assistance of the 3D InceptionResNetV2 model: the average junior radiologists’ accuracy increased by 16.87%, and senior radiologists’ accuracy increased by 11.12%. This indicates that the InceptionResNetV2 model can effectively help radiologists improve diagnostic accuracy and efficiency, especially significantly aiding the diagnostic abilities of junior doctors, and providing crucial support for clinical decision-making.

In terms of visualization capabilities, the DL model proposed in this study can automatically locate the nodule, enabling the visualization of the nodule. Grad-CAM highlights key areas of the thyroid calcified nodules, helping doctors understand why the model made certain diagnoses, thus enhancing the interpretability of the deep learning model. The DeLong test shows a statistically significant difference between the 3D and 2D models (*P* < 0.05), indicating that the 3D model demonstrates superior performance in predicting the malignancy of thyroid calcified nodules—especially for early-stage PTC with subtle calcifications.

The five DL networks used in this study each have their own advantages. InceptionResNetV2 combines the Inception module with residual learning, allowing for the extraction of multi-scale features (1 × 1, 3 × 3, 5 × 5 convolutions) while reducing computational costs by 30% compared to standard 3D models [31]; Xception significantly reduces parameters and improves efficiency through depthwise separable convolutions [32]; InceptionV3 is designed with multiple convolution kernel sizes, adapting to diverse inputs [33]; DenseNet121 alleviates the vanishing gradient problem through dense connections, enhancing feature reuse [34]; and ResNet50 addresses the degradation problem in deep networks through its residual learning structure [35]. These five models each have unique characteristics in ultrasound video analysis, effectively improving the accuracy and efficiency of nodule classification.

Although this study demonstrates the advantages of deep learning models in predicting the malignancy of thyroid calcified nodules, there are still some limitations. First, as this is a retrospective study, there may be selection bias, and the data are limited to six hospitals, which could affect the generalizability of the model. Second, although dynamic videos outperform static images in terms of prediction accuracy, this technology requires higher equipment standards and specialized personnel, which may not be feasible in certain clinical settings. Additionally, the integration of other imaging features (such as color Doppler ultrasound, CT, MRI) or genomic data could be explored to further enhance predictive performance. With the development of AI technology, more intelligent and automated diagnostic systems may be achieved in the future, providing more precise support for clinical decision-making.

## Conclusions

This study shows that deep learning models, particularly the 3D InceptionResNetV2 model based on dynamic ultrasound videos (with calcification attention module and 3D Grad-CAM interpretability), can effectively predict the malignancy of thyroid calcified nodules and provide more comprehensive clinical information than static images. This technology shows potential for a more accurate and convenient auxiliary tool for the clinical evaluation of thyroid nodules, offering a scientific basis for the formulation of personalized treatment plans.

## Data Availability

The datasets generated and/or analysed during the current study are not publicly available due to privacy but are available from the corresponding author upon reasonable request.

## Abbreviations

ACC: Accuracy
AUC: Area under the receiver operating characteristic curve
CIs: Confidence intervals
CNN: Convolutional Neural Network
DL: Deep learning
NPV: Negative predictive value
PPV: Positive predictive value
ROI: Region of interest
SEN: Sensitivity
SPE: Specificity
US: Ultrasonography
FNA: Fine-needle aspiration
AI: Artificial intelligence
F1: F1 score
